# Regional and socioeconomic inequalities and factors associated with low birth weight in Ghana: a multilevel analysis of the 2022 Ghana Demographic and Health Survey

**DOI:** 10.3389/fped.2026.1846424

**Published:** 2026-10-02

**Authors:** Samuel Antwi, Mabel Pokua Amoako-Boateng, John Kuumuori Ganle, Kofi Agyabeng, Godwin Adzakpah, Morrison Asiamah, Welbeck Amoani Twum, Victor Luckyboy Dzramado, Rebecca Ofosu, Eric Koka

**Affiliations:** 1 School of Public Health, University of Ghana, Accra, Ghana; 2 Department of Health Information Management, School of Allied Health Sciences, University of Cape Coast, Cape Coast, Ghana; 3 School of Medical Sciences, University of Cape Coast, Cape Coast, Ghana; 4 Department of Biostatistics, Cape Coast Teaching Hospital, Cape Coast, Ghana; 5 Department of Health Information Management, Cape Coast Metropolitan Hospital, Cape Coast, Ghana; 6 Department of Sociology and Anthropology, University of Cape Coast, Cape Coast, Ghana

**Keywords:** Demographic and Health Survey (DHS), determinants, Ghana, low birth weight, regional disparities, socioeconomic inequalities

## Abstract

**Introduction:**

Low birth weight (LBW) remains a major public health challenge globally, particularly in low- and middle-income countries (LMICs). In Ghana, socioeconomic and regional inequalities in birth outcomes persist. This study examined LBW prevalence, socioeconomic and regional inequalities, and factors associated with LBW using nationally representative data.

**Methods:**

A secondary analysis of the 2022 Ghana Demographic and Health Survey (GDHS) was conducted using a weighted sample of 5,768 live births to women aged 15–49 years. Multilevel binary logistic regression was used to assess factors associated with LBW, with a random intercept specified at the DHS cluster level. Socioeconomic inequality was assessed using a concentration curve ranked by household wealth. Regional differences were described using regional prevalence estimates, while between-cluster variation was assessed using the intraclass correlation coefficient (ICC) and median odds ratio (MOR).

**Results:**

The overall prevalence of LBW was 11.0%. Prevalence was highest among births in the poorest and poorer wealth quintiles (13%) and lowest in the richest quintile (6%). Regionally, prevalence was highest in Ashanti (15%) and Northern (14%) and lowest in Greater Accra (6%). In the fully adjusted multilevel model (*n* = 3,421), multiple pregnancy was associated with substantially higher odds of LBW (AOR = 10.09, 95% CI: 6.19–16.44), while adequate ANC attendance (≥8 visits) was associated with lower odds of LBW (AOR = 0.46, 95% CI: 0.32–0.66). The association between household wealth and LBW was not statistically significant after adjustment. Cluster-level ICC was 11.1% in the final model, indicating meaningful between-cluster variation.

**Conclusions:**

LBW prevalence varied across socioeconomic and regional groups in Ghana. Multiple pregnancy and adequate ANC attendance were the principal factors associated with LBW in the fully adjusted model, while household wealth was not statistically significant after adjustment. The findings support strengthening quality ANC, particularly for pregnancies at higher risk, alongside equity-oriented maternal and newborn health services.

## 1 Introduction

Birth weight is a fundamental indicator of newborn health, reflecting both the intrauterine environment and broader socioeconomic conditions affecting mothers (1). Low birth weight (LBW), defined as less than 2.5 kg at birth, is associated with increased risks of neonatal mortality, morbidity, impaired cognitive development, and chronic diseases later in life (2). According to recent WHO and UNICEF estimates, approximately 15% of live births globally are LBW, with the vast majority occurring in low- and middle-income countries (LMICs) where poverty, limited healthcare access, and maternal undernutrition remain prevalent (1). These disparities demonstrate how inequalities in birth outcomes are shaped by intersecting factors such as wealth, education, healthcare access, and geographic location (3).

In sub-Saharan Africa (SSA), LBW remains a major contributor to neonatal mortality and adverse child health outcomes (4). Current estimates suggest that approximately 13%–15% of births in SSA are LBW, with marked disparities across and within countries (1, 5). Higher prevalence has been reported among adolescents, rural populations, and economically disadvantaged households, reflecting persistent inequities in maternal nutrition and access to quality healthcare (2, 6). Previous studies have identified maternal education, household wealth, antenatal care (ANC) utilisation, and place of residence as important factors associated with LBW across LMICs (7). However, much of the existing literature has focused primarily on individual-level factors, with limited attention to contextual and community-level influences on birth outcomes.

Ghana mirrors many of these regional patterns. Recent nationally representative data from the 2022 Ghana Demographic and Health Survey (GDHS) indicate that approximately 11%–12% of live births are LBW, with disparities persisting across regions and socioeconomic groups (8). Spatial analyses consistently report higher concentrations of LBW in the Northern and middle zones compared with coastal regions such as Greater Accra (9). Previous Ghanaian studies have linked LBW to maternal education, household wealth, antenatal care utilisation, and place of residence (2). Biological and behavioural factors, including maternal age, parity, pre-pregnancy body mass index, and gestational weight gain, have also been associated with variations in birth weight (10–12).

Low birth weight remains a priority under the Sustainable Development Goals (SDGs), particularly SDG 3, due to its close relationship with neonatal mortality and long-term child health outcomes (13). Ghana has implemented several maternal and newborn health interventions, including the Free Maternal Health Care Policy under the National Health Insurance Scheme (NHIS), iron and folic acid supplementation programmes, and intermittent preventive treatment for malaria in pregnancy (14). Despite these efforts, progress in reducing LBW has remained slow and uneven, with persistent socioeconomic and regional inequalities highlighting the need for more context-specific evidence. Therefore, this study used a multilevel analytical approach to examine socioeconomic and contextual inequalities and factors associated with LBW in Ghana using nationally representative GDHS 2022 data.

## 2 Materials and methods

### 2.1 Study design, data sources and population

This study utilised data from the 2022 Ghana Demographic and Health Survey (GDHS), a nationally representative survey of women aged 15–49 years. The DHS is a standardised survey conducted across low- and middle-income countries to provide reliable estimates of maternal and child health indicators. The design incorporated a stratified two-stage cluster sampling approach to ensure representativeness across Ghana's regions and urban–rural strata. Detailed descriptions of the survey methodology are available in the official report (15).

The full women's dataset comprised 39,305 respondents. The analytic population for this study comprised 5,768 live births with reported birth weight, representing the weighted population used for descriptive estimates. DHS sampling weights were applied to obtain population-representative estimates.

### 2.2 Data access and management

Data for this study were obtained through a formal request to ICF, the custodian of the Demographic and Health Surveys (DHS) Program. Upon approval, the anonymised 2022 Ghana Demographic and Health Survey (GDHS) dataset was accessed and cleaned. The survey design was declared using the DHS woman's sampling weight (v005), primary sampling unit/cluster (v021), and strata (v022). Design-based descriptive estimates used the survey weights, PSU, and strata. The multilevel models were estimated separately using Stata's mixed-effects logistic regression framework, with the DHS sampling weight incorporated and a random intercept for DHS cluster; therefore, the “svy:” prefix was not applied to the mixed-effects likelihood model. This distinction is made explicit to avoid implying that the full design-based survey variance estimator was directly applied to the random-effects model (Supplementary File 4).

### 2.3 Study variables and measurement

The outcome variable for this study was low birth weight (LBW), defined as a birth weight less than 2,500 g, coded as 1, while normal birth weight (≥2,500 g) was coded as 0, consistent with WHO's global definition (1). Independent variables were selected based on previous literature and grouped into biological and demographic factors, socioeconomic factors, and community-level factors.

Biological and demographic factors included maternal age, birth order, birth interval, pregnancy type, and the number of children ever born. Birth interval (*n* = 4,452) was categorised as Short, Optimal, and Long. Pregnancy type was coded as Singleton (0) and Multiple (1). The number of children ever born was categorised as 1 child, 2–3 children, 4–5 children, 6–7 children, and ≥8 children.

Socioeconomic factors comprised maternal education, categorized as No education, Primary, Secondary, and Higher; household wealth index, grouped into five quintiles (Poorest, Poorer, Middle, Richer, and Richest); employment status, coded as No (0) and Yes (1); and marital status, categorized as Never in union, Married, living with a partner, Widowed, Divorced, and Separated.

Community-level factors included place of residence, coded as Urban (0) and Rural (1), and region of residence, categorised according to the GDHS regional classification (15).

Additional maternal health indicators were also included. Antenatal care (ANC) visits were categorised as Inadequate (<8 visits) and Adequate (≥8 visits). Wanted pregnancy (*n* = 5,268) was categorised as Then, Later, and No more. Iron supplementation during pregnancy (*n* = 5,218) was coded as No (0) and Yes (1).

### 2.4 Data management

Data from the 2022 GDHS were cleaned and prepared before analysis. Complete-case analysis was used for the multivariable models: observations with missing values on variables included in a given model were excluded from that model. No imputation was performed. The fully adjusted model was based on 3,421 complete observations. Because birth interval had 22.8% missing data and iron supplementation had 9.5% missing data, complete-case analysis may introduce selection bias if missingness is related to LBW or other study characteristics (Supplementary File 2).

### 2.5 Statistical analysis

All analyses were conducted using Stata version 17. Descriptive statistics were estimated using the DHS sampling weights and the complex survey design (PSU and strata). Covariates were specified *a priori* using an epidemiological/conceptual framework informed by established pathways and previous evidence rather than relying solely on statistical significance in bivariate analyses. Multilevel binary logistic regression models were then used to estimate adjusted associations.

The analytical hierarchy was two-level rather than three-level: individual births were the level-1 observations and DHS sampling clusters/communities were the level-2 units. Although births can be linked to households in DHS data, a separate household-level random intercept was not included in the fitted models. Household characteristics were entered as fixed-effect covariates where applicable. A random intercept for DHS cluster was used to account for residual between-cluster heterogeneity.

Four models were estimated sequentially. The null model (Model 0) was fitted without explanatory variables to assess baseline cluster-level variance. Model I incorporated biological and demographic factors, Model II added socioeconomic factors, and Model III was the fully adjusted model. Random-intercept variance, ICC, and MOR were calculated to quantify between-cluster heterogeneity. Model fit was evaluated using deviance (−2 log likelihood), AIC, and BIC, with lower values indicating better model fit. Adjusted odds ratios (AORs) with 95% confidence intervals (CIs) were reported.

Effect modification was assessed using selected, theoretically informed interaction terms within the multilevel mixed-effects logistic regression models. The primary interaction of interest was wealth index by place of residence, given its relevance in capturing socioeconomic and geographic inequalities in low birth weight (Supplementary File 3).

Socioeconomic inequality was assessed using a concentration curve ranking observations by household wealth index. A regional concentration curve was not retained because administrative regions are categorical geographic units without a natural socioeconomic ranking. Regional inequality was instead described using region-specific LBW prevalence, while between-cluster variation was quantified using the ICC and MOR from the multilevel models.

Multicollinearity among independent variables was assessed using the Variance Inflation Factor (VIF) (Supplementary File 1). Variables with high VIF values were considered in relation to their conceptual importance and overlap with other covariates. Maternal age and parity were retained because of their established epidemiological relevance and their role in the conceptual model. The mean VIF was 4.04.

This study used secondary, de-identified data from the 2022 GDHS. The 2022 GDHS survey protocol received ethical clearance from the Ethical Review Committee of the Ghana Health Service and the ICF Institutional Review Board. The authors obtained permission to access and analyse the anonymised dataset through the DHS data-access process (15).

## 3 Results

### 3.1 Background characteristics of the study respondents

Table 1 presents the weighted background characteristics of the 5,768 live births included in the descriptive analysis.

**Table 1:** Background characteristics of the study respondents (weighted percentages; *N* = 5,768).

Variables	Category	Weighted frequency *N* (%)
Maternal age	15–19 years	288 (5.5)
	20–24 years	1,074 (20.5)
	25–29 years	1,282 (24.5)
	30–34 years	1,241 (23.7)
	35–49 years	1,183 (25.8)
Marital status	Never in union	656 (11.4)
	Married	3,518 (61.1)
	Living with a partner	1,276 (22.2)
	Widowed	58 (1.0)
	Divorced	46 (0.8)
	No longer together	204 (3.5)
Place of residence	Urban	2,756 (47.8)
	Rural	3,012 (52.2)
Religion	Christian	4,025 (69.8)
	Islam	1,436 (24.9)
	Traditional/Spiritualist	173 (3.0)
	No religion/Other	120 (2.3)
Currently working	No	1,310 (22.7)
	Yes	4,458 (77.3)
Educational status	No education	1,264 (21.9)
	Primary	889 (15.4)
	Secondary	3,094 (53.6)
	Higher	521 (9.0)
Wealth quintile	Poorest	1,372 (23.8)
	Poorer	1,209 (21.0)
	Middle	1,148 (19.9)
	Richer	1,077 (18.7)
	Richest	962 (16.6)
Children ever born	1 child	1,436 (24.9)
	2–3 children	2,203 (38.2)
	4–5 children	1,332 (23.1)
	6–7 children	611 (10.6)
	≥8 children	186 (3.2)
ANC visits	Inadequate	3,784 (64.6)
	Adequate	1,984 (35.4)
Household size	1–4 members	2,262 (39.3)
	5–8 members	2,775 (48.2)
	≥9 members	731 (12.5)
Birth order	First	1,303 (22.6)
	Low/Medium	2,890 (50.1)
	High	1,575 (27.3)
Types of birth	Singleton	5,474 (94.9)
	Multiple	294 (5.1)
Birth interval (*n* = 4,452)	Short	882 (19.8)
	Optimal	2,691 (60.5)
	Long	879 (19.7)
Wanted pregnancy	Then	3,167 (61.4)
	Later	1,504 (29.3)
	No more	597 (9.3)
Iron supplementation (*n* = 5,218)	No	350 (7.3)
	Yes	4,408 (92.7)

Percentages are weighted to account for the complex survey design. The total weighted sample size is *N* = 5,768. However, denominators vary across variables due to item-specific missing data, as some respondents had incomplete information for certain covariates. Therefore, valid percentages for each variable are based on available responses for that specific variable and may differ from the total sample size.

Most women were married (61.1%), and 22.2% were living with a partner, whereas 11.4% had never been in union. Small proportions were widowed (1.0%), divorced (0.8%), or no longer together (3.5%). Slightly more than half of the respondents resided in rural areas (52.2%), with 47.8% living in urban settings. Regarding religion, the majority were Christians (69.8%), followed by Muslims (24.9%), while 3.0% practiced traditional/spiritualist religions and 2.3% reported no religion or other affiliations. A substantial proportion of women were currently working (77.3%).

In terms of education, over half had attained secondary education (53.6%), 21.9% had no formal education, 15.4% had primary education, and 9.0% had higher education. Wealth distribution was relatively even across quintiles, though slightly higher among the poorest (23.8%) and poorer (21.0%) groups compared to the richest (16.6%).

Concerning fertility characteristics, 38.2% of women had two to three children, while 24.9% had one child. Approximately 23.1% had four to five children, 10.6% had six to seven children, and 3.2% had eight or more children. Most respondents reported inadequate antenatal care (ANC) visits (64.6%), while 35.4% had adequate ANC attendance.

Half of the births were of low to medium birth order (50.1%), while 27.3% were high birth order and 22.6% were first births. The vast majority of births were singletons (94.9%), while 5.1% were multiple births. Among women with available birth interval data (*n* = 4,452), most had optimal birth intervals (60.5%), while 19.8% and 19.7% had short and long intervals, respectively.

Regarding pregnancy intention, 61.4% of pregnancies were wanted at the time, 29.3% were wanted later, and 9.3% were not wanted. Among women with data on iron supplementation (*n* = 5,218), 92.7% reported receiving iron supplementation during pregnancy.

### 3.2 Weighted distribution of birth weight

Figure 1 presents the weighted distribution of birth weight among the study population (*n* = 5,768). Normal birth weight accounted for 89.0% of births, while 11.0% were classified as LBW (<2,500 g).

**Figure 1:**
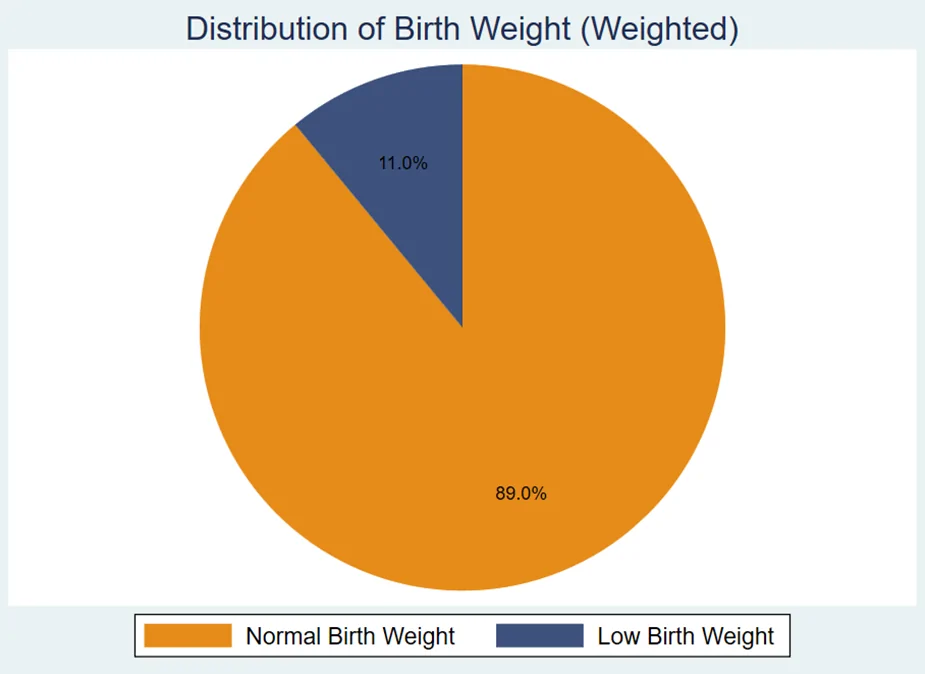
Distribution of birth weight in Ghana, 2022.

### 3.3 Prevalence of low birth weight by wealth quintiles and region

Figure 2 presents LBW prevalence across wealth quintiles. Prevalence was 13% in both the poorest and poorer quintiles, 12% in the middle quintile, 11% in the richer quintile, and 6% in the richest quintile.

**Figure 2:**
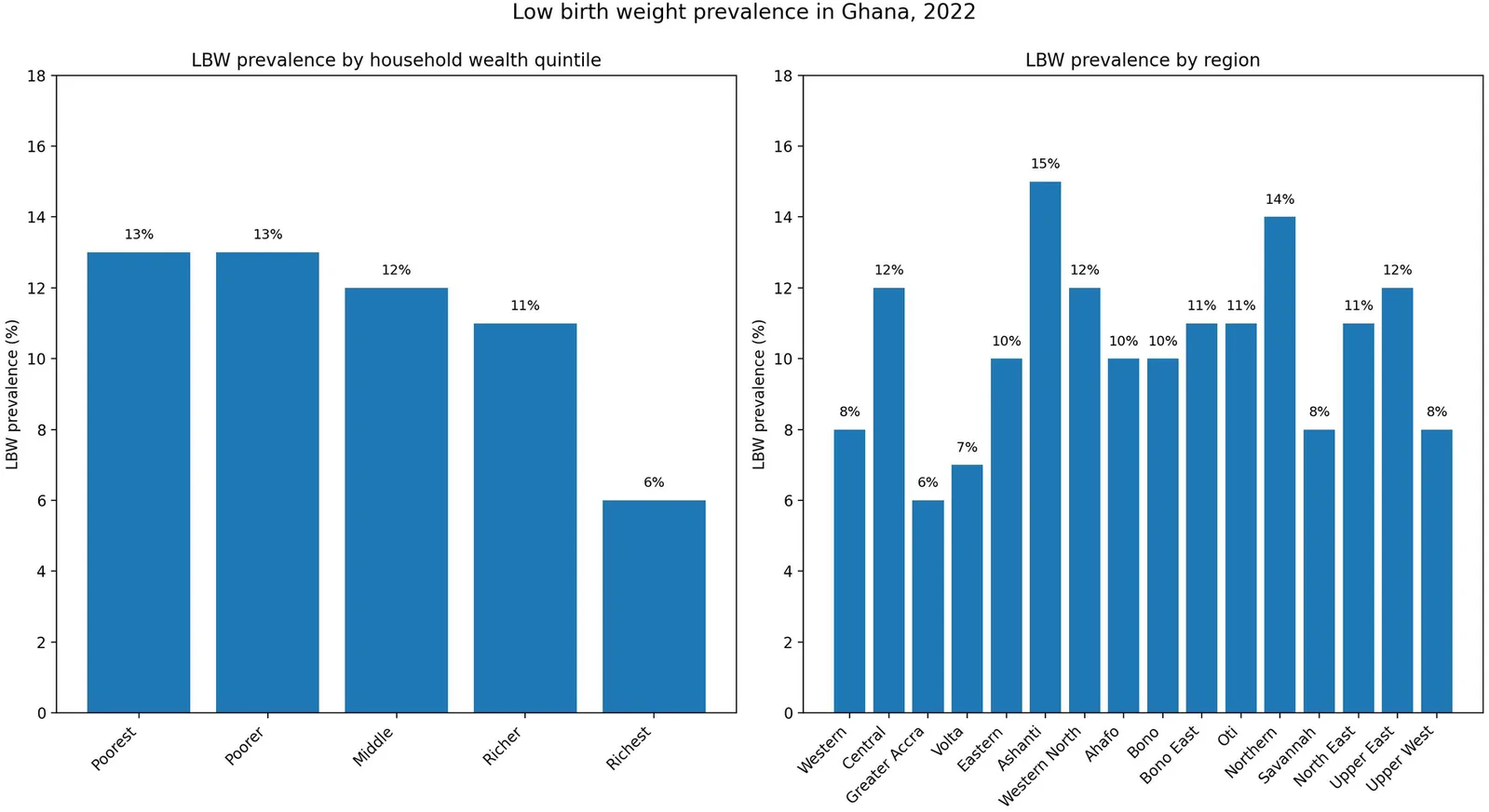
Prevalence of low birth weight by household wealth quintile and region, Ghana, 2022.

Regional variation in LBW prevalence. Prevalence was highest in Ashanti (15%) and Northern (14%), followed by Central, Western North, and Upper East (12% each). Greater Accra had the lowest prevalence (6%), followed by Volta (7%), while Western, Savannah, and Upper West each recorded 8%. These estimates describe regional differences and are not interpreted as a concentration-based socioeconomic ranking.

### 3.4 Socioeconomic and regional inequality in low birth weight

Figure 3 presents the concentration curve for LBW ranked by household wealth. The curve indicates a pro-poor concentration of LBW, consistent with the observed gradient in prevalence across wealth quintiles. Because regions are categorical geographic units without an inherent socioeconomic ranking, the former regional concentration curve was removed. Regional inequality is instead presented using the regional prevalence estimates with the multilevel assessment of between-cluster variation.

**Figure 3:**
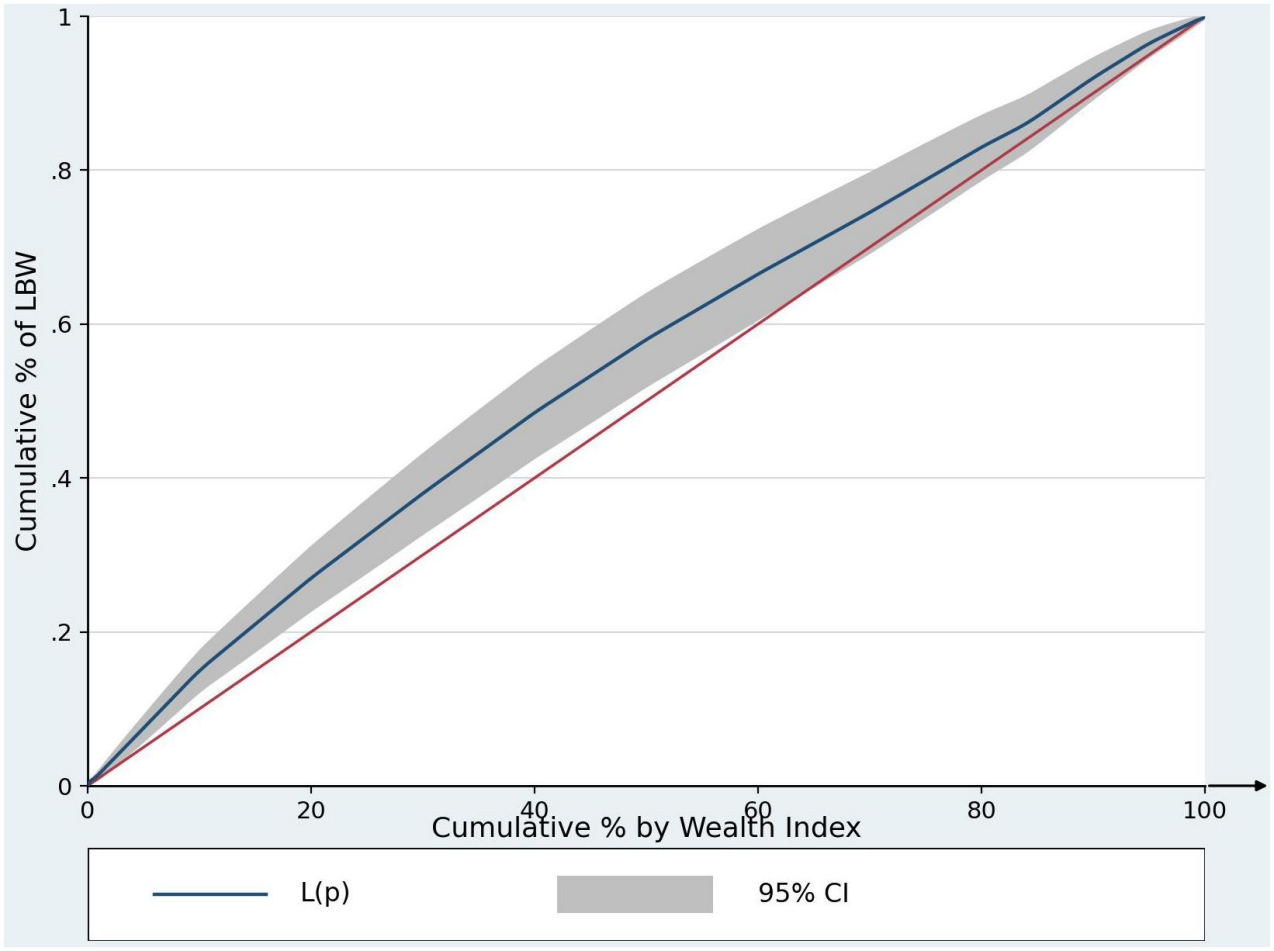
Concentration curve for LBW ranked by wealth in Ghana.

### 3.5 Random effects and model fit analysis

Table 2 presents the multilevel random-effects results, including random-intercept variance, cluster-level ICC, MOR, and model fit statistics. The number of observations was 4,496 in the null and Model II and 3,421 in Models I and III; the number of clusters was 616 and 607, respectively. The final model had a cluster-level ICC of 0.111 and an MOR of 1.82, indicating meaningful residual between-cluster heterogeneity.

**Table 2:** Random effects, MOR, and model fit statistics from multilevel logistic regression models for low birth weight in Ghana, 2022.

Parameter	Null model	Model I	Model II	Model III
Random intercept variance	0.402	0.571	0.324	0.411
Cluster-level ICC	0.109	0.148	0.090	0.111
Median Odds Ratio (MOR)	1.83	2.05	1.72	1.82
Log likelihood	−1,494.09	−958.57	−1,468.71	−939.87
Deviance (−2 log likelihood)	2,988.18	1,917.14	2,937.42	1,879.73
AIC	2,992.18	1,945.14	3,005.42	1,969.74
BIC	3,005.00	2,031.07	3,223.40	2,245.93
Number of observations	4,496	3,421	4,496	3,421
Number of groups	616	607	616	607

AIC, Akaike information criterion; BIC, Bayesian information criterion; ICC, intraclass correlation coefficient; MOR, median odds ratio.

The median odds ratio (MOR) further quantified this heterogeneity, ranging from 1.72 in Model II to 2.05 in Model I, indicating moderate but meaningful between-cluster variation in the odds of LBW across all models. The MOR consistently exceeded 1.5 across all models, confirming meaningful residual heterogeneity between clusters even after adjustment for covariates.

The ICC increased from the null model (0.109) to Model I (0.148), reflecting changes in the distribution of unexplained variance after adjustment for individual-level biological factors. This pattern may occur when the inclusion of covariates alters the partitioning of variance between individual and cluster levels rather than indicating model misspecification. However, subsequent models showed a reduction and stabilisation of ICC values, suggesting that the inclusion of socioeconomic and contextual variables improved model specification. The MOR results corroborated this interpretation by consistently showing non-trivial cluster-level heterogeneity across all models, providing a more intuitive measure of contextual influence than ICC alone.

Log-likelihood improved from −1,494.09 in the null model to −939.87 in Model III, while deviance (−2 log likelihood) decreased from 2,988.18 to 1,879.73. AIC decreased from 2,992.18 to 1,969.74 and BIC decreased from 3,005.00 to 2,245.93. Model III was retained as the final model based on the reported fit statistics.

#### 3.5.1 Factors associated with low birth weight

To identify factors associated with LBW in Ghana, multivariable multilevel binary logistic regression was performed. The candidate variables were prespecified using an epidemiological/conceptual framework and prior evidence. In the fully adjusted model, pregnancy type and ANC attendance were statistically associated with LBW, whereas household wealth was not statistically significant after adjustment.

Neonates from multiple pregnancies had substantially higher odds of LBW than singleton births (AOR = 10.09, 95% CI: 6.19–16.44). Adequate ANC attendance (≥8 visits) was associated with lower odds of LBW compared with inadequate attendance (AOR = 0.46, 95% CI: 0.32–0.66). The richest wealth quintile had lower point-estimate odds than the poorest quintile, but the association was not statistically significant (AOR = 0.60, 95% CI: 0.27–1.35). Thus, the point estimate suggested lower odds of LBW among women in the richest wealth quintile, although the confidence interval included the null value and therefore did not provide strong statistical evidence of an association.

An interaction analysis was further conducted to assess potential effect modification between household wealth index and place of residence on LBW. The interaction model showed significant interaction effects for rural residence among mothers in the middle- and richer-wealth categories, suggesting heterogeneity in the relationship between socioeconomic status and LBW across residence settings (Supplementary File 3).

Other maternal and household characteristics, including maternal age, marital status, maternal education, religion, employment status, parity, birth interval, residence, and region, were not statistically associated with LBW after adjustment (Table 3), although short birth spacing has been associated with adverse birth outcomes in other settings (24).

**Table 3:** Multivariable multilevel binary logistic regression analysis of factors associated with LBW in Ghana (GDHS 2022).

Variables	Model I AOR (95% CI)	Model II AOR (95% CI)	Model III AOR (95% CI)
Maternal age			
15–19	Ref	–	Ref
20–24	0.63 (0.18–2.27)	–	0.60 (0.16–2.21)
25–29	0.71 (0.21–2.45)	–	0.76 (0.21–2.74)
30–34	0.59 (0.17–2.09)	–	0.73 (0.19–2.82)
35–49	0.49 (0.14–1.76)	–	0.68 (0.18–2.51)
Total children ever born			
1 child	Ref	–	Ref
2–3 children	0.79 (0.40–1.57)	–	0.87 (0.40–1.87)
4–5 children	0.69 (0.32–1.49)	–	0.70 (0.29–1.69)
6–7 children	1.04 (0.42–2.58)	–	0.93 (0.34–2.54)
≥8 children	0.94 (0.30–2.92)	–	0.97 (0.28–3.31)
Birth interval			
Short	Ref	–	Ref
Optimal	1.07 (0.71–1.63)	–	0.96 (0.62–1.47)
Long	1.41 (0.82–2.42)	–	1.31 (0.79–2.18)
Pregnancy type			
Singleton	Ref	–	Ref
Multiple	9.91 (6.00–16.37)	–	10.09 (6.19–16.44)
ANC visits			
Inadequate (<8 visits)	Ref	–	Ref
Adequate (≥8 visits)	0.44 (0.31–0.62)	–	0.46 (0.32–0.66)
Wealth index			
Poorest	–	Ref	Ref
Poorer	–	0.83 (0.57–1.21)	0.84 (0.50–1.40)
Middle	–	0.69 (0.44–1.10)	0.93 (0.56–1.55)
Richer	–	0.63 (0.35–1.14)	0.88 (0.44–1.74)
Richest	–	0.36 (0.18–0.72)	0.60 (0.27–1.35)
Maternal education			
No education	–	Ref	Ref
Primary	–	1.19 (0.73–1.95)	1.20 (0.68–2.13)
Secondary	–	1.24 (0.85–1.81)	1.24 (0.75–2.05)
Higher	–	1.48 (0.81–2.70)	1.24 (0.53–2.90)
Employment			
Not working	–	Ref	Ref
Working	–	0.84 (0.63–1.13)	0.99 (0.64–1.52)
Marital status			
Never in union	–	Ref	Ref
Married	–	1.19 (0.72–1.98)	0.72 (0.34–1.51)
Cohabiting	–	1.69 (0.96–2.95)	1.01 (0.49–2.07)
Widowed	–	1.10 (0.17–7.00)	1.06 (0.14–8.06)
Divorced	–	0.32 (0.04–2.58)	0.29 (0.03–2.49)
Separated	–	0.78 (0.30–2.01)	0.51 (0.13–2.03)
Religion			
Christian	–	Ref	Ref
Muslim	–	1.02 (0.72–1.44)	1.04 (0.69–1.56)
Traditional	–	0.74 (0.27–1.98)	0.79 (0.26–2.44)
None/other	–	1.04 (0.34–3.26)	0.86 (0.31–2.37)
Residence			
Urban	–	Ref	Ref
Rural	–	0.88 (0.64–1.22)	0.98 (0.68–1.41)
Region			
Western	–	Ref	Ref
Central	–	1.44 (0.67–3.09)	1.39 (0.59–3.27)
Greater Accra	–	0.78 (0.33–1.83)	0.71 (0.28–1.80)
Volta	–	0.71 (0.31–1.61)	0.63 (0.25–1.55)
Eastern	–	1.25 (0.58–2.69)	1.61 (0.71–3.64)
Ashanti	–	1.98 (0.99–3.96)	1.89 (0.84–4.24)
Western North	–	1.36 (0.63–2.91)	1.02 (0.44–2.41)
Ahafo	–	0.95 (0.43–2.12)	0.75 (0.30–1.85)
Bono	–	1.21 (0.56–2.62)	1.37 (0.56–3.33)
Bono East	–	1.22 (0.58–2.55)	1.30 (0.56–3.01)
Oti	–	1.19 (0.52–2.74)	1.21 (0.49–2.99)
Northern	–	1.74 (0.80–3.77)	2.36 (0.98–5.66)
Savannah	–	0.78 (0.35–1.76)	0.74 (0.30–1.79)
North East	–	1.12 (0.50–2.55)	1.12 (0.45–2.76)
Upper East	–	1.28 (0.57–2.86)	1.31 (0.54–3.20)
Upper West	–	0.77 (0.35–1.71)	0.90 (0.39–2.09)

Values are adjusted odds ratios (AORs) with 95% confidence intervals (CIs) from multilevel multivariable binary logistic regression. “Ref” denotes the reference category. LBW is defined as birth weight <2,500 g. Model I includes biological/demographic factors, Model II adds socioeconomic factors, and Model III is the fully adjusted model. Model III is based on 3,421 complete observations. Descriptive frequencies were omitted from this regression table because the available frequency denominators were inconsistent across covariates. Statistical significance was assessed at *p* < 0.05.

## 4 Discussion

### 4.1 Prevalence and patterns of low birth weight

This study found an overall LBW prevalence of 11.0% in Ghana, with LBW unevenly distributed across socioeconomic and regional groups. This prevalence is broadly consistent with estimates from sub-Saharan Africa (4, 9, 20). National DHS analyses also report geographic clustering of LBW in northern and middle zones (2). The observed differences indicate that national averages may mask important regional and socioeconomic variation in birth outcomes.

The clustering of LBW in northern and middle zones highlights geographic and socioeconomic differences in maternal and newborn health conditions. Studies from other settings have identified different combinations of maternal, reproductive, and contextual factors associated with LBW (21, 23, 25). The present findings therefore support context-specific interpretation rather than assuming a common causal pathway across countries.

### 4.2 Socioeconomic inequalities

A clear socioeconomic gradient in low birth weight (LBW) was observed, with the highest prevalence among mothers in the poorest wealth quintile and the lowest among the richest. This finding is consistent with prior Ghanaian studies showing that poorer households face higher odds of LBW (2, 10, 11) and with regional evidence from Kenya and Tanzania, where similar socioeconomic gradients have been reported (16, 17). Systematic reviews further confirm that higher maternal education reduces LBW risk across low- and middle-income countries (7), while meta-analyses indicate that adequate antenatal care (ANC) attendance substantially lowers the odds of LBW (18). Poorer women may experience food insecurity, delayed care-seeking, and barriers to accessing timely maternal healthcare services, all of which may adversely affect pregnancy outcomes (22, 26).

Although wealth was associated with LBW in the descriptive analysis, it was not statistically significant in the fully adjusted model. The point estimate suggested lower odds of LBW among women in the richest wealth quintile (AOR = 0.60), although the confidence interval included the null value and therefore did not provide strong statistical evidence of an association. The descriptive socioeconomic gradient nevertheless supports continued attention to equity in maternal and newborn health services.

#### 4.2.1 Regional disparities

Regional prevalence varied substantially, with the highest estimates in Ashanti and Northern and the lowest in Greater Accra. These differences may reflect variation in healthcare access, maternal nutrition, transportation, and broader socioeconomic conditions, but the cross-sectional design does not permit causal attribution.

The regional findings should be interpreted as descriptive differences rather than as evidence of a ranked regional inequality gradient. Geographic variation may reflect multiple overlapping contextual factors, and the multilevel model provides additional evidence of residual between-cluster heterogeneity.

### 4.3 Factors associated with LBW

Pregnancy type and adequate ANC attendance were statistically associated with LBW in the fully adjusted model, while household wealth was not statistically significant after adjustment. Multiple pregnancy was strongly associated with higher odds of LBW, consistent with evidence from other African settings linking multiple gestations with preterm birth and reduced fetal growth (12). Adequate ANC attendance was associated with lower odds of LBW, consistent with studies from Ghana and Tanzania (17, 19).

The higher odds of LBW among multiple pregnancies are biologically plausible given intrauterine competition and placental factors. The association between adequate ANC attendance and lower odds of LBW may reflect differences in access to screening, prevention, treatment, nutritional support, and timely referral; however, these mechanisms cannot be established from the present cross-sectional analysis. Although the wealth association was not statistically significant in the final model, the point estimate was below one and should be interpreted cautiously.

The interaction analysis suggested that the association between household wealth and LBW differed by place of residence, with significant interaction terms for rural residence among the middle- and richer-wealth categories. These findings indicate heterogeneity in the observed wealth–LBW association across residential settings, but they should not be interpreted as evidence of a causal effect.

These findings imply that strengthening the quality and content of ANC services is essential, particularly for women with multiple pregnancies who require specialised monitoring and nutritional support. Policies should continue to mitigate socioeconomic disparities by ensuring universal access to quality maternal health services, while further research should explore whether existing interventions have effectively reduced traditional socioeconomic and regional inequities in LBW outcomes.

#### 4.3.1 Random effects and contextual factors

Community-level factors accounted for a substantial proportion of the variation in LBW, with ICC values ranging from 9.0% to 14.8% across the reported models and 11.1% in the final model. The MOR also indicated meaningful residual between-cluster heterogeneity. These findings suggest that contextual differences between DHS clusters are relevant to LBW patterns even after adjustment for measured individual and household characteristics.

## 5 Strengths and limitations

A key strength of this study is the use of nationally representative 2022 GDHS data and multilevel modelling to quantify residual between-cluster heterogeneity. The study also examined both socioeconomic inequality and regional variation, providing complementary perspectives on LBW in Ghana.

Several limitations should be considered. First, the cross-sectional nature of the GDHS limits temporal ordering and prevents causal inference; associations should therefore not be interpreted as causal determinants of low birth weight. Second, gestational age was not adequately available for incorporation into the final analysis. Because LBW combines prematurity and fetal growth restriction, the inability to distinguish these pathways is an important limitation and may contribute to residual confounding. Third, the DHS 2022 birth-weight indicator includes births for which birth weight was available from either a written record or the mother's report; the present analysis did not stratify estimates by source of measurement or timing of measurement, and therefore measurement error and differential reporting cannot be excluded (15). Fourth, women with a documented/reported birth weight may differ from women whose births lacked birth-weight information, creating potential selection bias.

Fifth, important biological and clinical factors, including maternal BMI/nutritional status, maternal complications, prematurity, and fetal growth restriction, were not adequately addressed in the final model. Their omission may result in residual confounding. Sixth, complete-case analysis reduced the sample to 3,421 observations in the fully adjusted model. Birth interval had 22.8% missing data and iron supplementation had 9.5% missing data. Because missingness may be related to participant characteristics or the outcome, complete-case analysis may introduce selection bias and reduce precision and generalisability. A comparison of included and excluded participants and a formal sensitivity analysis using multiple imputation were not performed and should be considered in future analyses. Finally, regional aggregation may mask substantial within-region heterogeneity.

## 6 Conclusion

This study found substantial socioeconomic and regional variation in LBW prevalence in Ghana, together with meaningful residual between-cluster heterogeneity. In the fully adjusted multilevel model, multiple pregnancy was associated with substantially higher odds of LBW, while adequate ANC attendance was associated with lower odds. Household wealth was not statistically significant after adjustment, although the point estimate for the richest quintile was below one. These findings support continued efforts to improve the quality and continuity of ANC, particularly for pregnancies at higher risk, while strengthening equitable access to maternal and newborn health services. Because of the cross-sectional design and important unmeasured factors such as gestational age and maternal nutritional status, the findings should be interpreted as associations rather than causal effects.

To address these disparities, targeted and intensified ANC services for high-risk pregnancies, particularly multiple gestations are recommended. This should include early identification, close monitoring, and adherence to the full schedule of recommended ANC visits. Strengthening delivery through Community-based Health Planning and Services (CHPS) compounds will enhance maternal nutrition counselling, risk screening, and continuity of care. Linking pregnant women in the poorest wealth quintiles to existing cash transfer and maternal support programmes is also essential, especially in northern and other underserved regions. Such measures would reduce financial barriers to ANC utilisation, improve maternal nutrition, and mitigate socioeconomic inequalities associated with LBW. A coordinated, equity-focused policy response combining strengthened high-risk ANC services and targeted social protection is crucial to reduce LBW disparities and advance Ghana's progress toward improved maternal and child health outcomes.

## Data Availability

The original contributions presented in the study are included in the article/Supplementary Material, further inquiries can be directed to the corresponding author.
