# Supplementary material for: Regional and socioeconomic inequalities and factors associated with low birth weight in Ghana: a multilevel analysis of the 2022 Ghana Demographic and Health Survey
Source: Front Pediatr. 2026 Oct 2;14:1846424. doi: 10.3389/fped.2026.1846424 (PMC13672600; doi:10.3389/fped.2026.1846424)
Supplement: Supplementary file 1 [file Supplementaryfile1.docx]

**Supplementary File 4**

*Analytical specification and survey design*

**Survey design specification.** The DHS sampling weight was generated as wt = v005/1,000,000, and the survey design was then declared using svyset v021 [pweight=wt], strata(v022). Design-based descriptive estimates were obtained using the declared DHS survey design. For the multilevel analysis, a mixed-effects logistic model with the DHS sampling weight and a random intercept for DHS cluster was used; the svy: prefix was not applied to the mixed-effects likelihood model. The fitted hierarchy therefore consisted of births at level 1 and DHS clusters at level 2.

**Illustrative Stata specification.**

melogit lbw <prespecified_covariates> [pweight=wt] || v021:

The exact variable list corresponds to the fully adjusted Model III reported in Table 3. Model-based 95% confidence intervals were reported from the mixed-effects logistic model.

**Reporting statement.** The manuscript was checked against the STROBE recommendations for observational studies, covering study design, participant selection, variables, bias, missing data, statistical methods, estimates, limitations, and interpretation.
