# Supplementary material for: Regional and socioeconomic inequalities and factors associated with low birth weight in Ghana: a multilevel analysis of the 2022 Ghana Demographic and Health Survey
Source: Front Pediatr. 2026 Oct 2;14:1846424. doi: 10.3389/fped.2026.1846424 (PMC13672600; doi:10.3389/fped.2026.1846424)
Supplement: Supplementary file 2 [file Table1.docx]

| **Supplementary File 1: Variance Inflation Factor (VIF) for Predictors of Low Birth Weight** | | |
| --- | --- | --- |
| **Variable** | **VIF** | **1/VIF** |
| **Maternal age** |  |  |
| 20–24 years | 12.11 | 0.083 |
| 25–29 years | 21.46 | 0.047 |
| 30–34 years | 24.27 | 0.041 |
| 35–49 years | 26.4 | 0.038 |
| **Children ever born** |  |  |
| 2–3 children | 5.87 | 0.17 |
| 4–5 children | 6.15 | 0.163 |
| 6–7 children | 4.21 | 0.238 |
| 8+ children | 2.09 | 0.479 |
| **Birth interval** |  |  |
| Optimal | 1.79 | 0.559 |
| Long | 1.81 | 0.552 |
| **Plurality** |  |  |
| Multiple birth | 1.06 | 0.94 |
| **ANC attendance** |  |  |
| ≥8 visits | 1.07 | 0.936 |
| **Wealth index** |  |  |
| Poorer | 1.67 | 0.599 |
| Middle | 2.07 | 0.483 |
| Richer | 2.37 | 0.422 |
| Richest | 2.85 | 0.351 |
| **Educational level** |  |  |
| Primary | 1.5 | 0.667 |
| Secondary | 2.19 | 0.457 |
| Higher | 1.93 | 0.519 |
| **Employment status** |  |  |
| Working | 1.08 | 0.929 |
| **Marital status** |  |  |
| Married | 5.17 | 0.193 |
| Living with partner | 4.06 | 0.246 |
| Widowed | 1.23 | 0.813 |
| Divorced | 1.16 | 0.864 |
| Separated | 1.51 | 0.66 |
| **Religion** |  |  |
| Islam | 1.53 | 0.656 |
| Traditional/Spiritualist | 1.09 | 0.919 |
| No religion/Other | 1.06 | 0.947 |
| **Place of residence** |  |  |
| Rural | 1.61 | 0.621 |
| **Region** |  |  |
| Central | 2.11 | 0.474 |
| Greater Accra | 2.07 | 0.482 |
| Volta | 1.85 | 0.542 |
| Eastern | 1.98 | 0.506 |
| Ashanti | 2.27 | 0.441 |
| Western North | 2.04 | 0.49 |
| Ahafo | 2.27 | 0.441 |
| Bono | 2.06 | 0.486 |
| Bono East | 2.59 | 0.386 |
| Oti | 2.15 | 0.465 |
| Northern | 3.2 | 0.313 |
| Savannah | 2.71 | 0.369 |
| North East | 3.2 | 0.312 |
| Upper East | 2.57 | 0.39 |
| Upper West | 2.49 | 0.402 |
| **Mean VIF** | **4.04** |  |

***All VIF values are below the commonly cited threshold of 10, indicating no significant multicollinearity***
