# Supplementary material for: Regional and socioeconomic inequalities and factors associated with low birth weight in Ghana: a multilevel analysis of the 2022 Ghana Demographic and Health Survey
Source: Front Pediatr. 2026 Oct 2;14:1846424. doi: 10.3389/fped.2026.1846424 (PMC13672600; doi:10.3389/fped.2026.1846424)
Supplement: Supplementary file 3 [file Table2.docx]

| **Supplement File 2. Missing Data Distribution for Key Independent Variables (GDHS 2022)** | | | | |
| --- | --- | --- | --- | --- |
| **Variable** | **Total sample (N)** | **Complete observations (n)** | **Missing (n)** | **Missing (%)** |
| Birth interval | 5,768 | 4,452 | 1,316 | 22.8 |
| Iron supplementation | 5,768 | 5,218 | 550 | 9.5 |
| Maternal age | 5,768 | 5,768 | 0 | 0 |
| Marital status | 5,768 | 5,768 | 0 | 0 |
| Place of residence | 5,768 | 5,768 | 0 | 0 |
| Religion | 5,768 | 5,768 | 0 | 0 |
| Currently working | 5,768 | 5,768 | 0 | 0 |
| Educational status | 5,768 | 5,768 | 0 | 0 |
| Wealth index | 5,768 | 5,768 | 0 | 0 |
| Children ever born | 5,768 | 5,768 | 0 | 0 |
| ANC visits | 5,768 | 5,768 | 0 | 0 |
| Household size | 5,768 | 5,768 | 0 | 0 |
| Birth order | 5,768 | 5,768 | 0 | 0 |
| Pregnancy intention | 5,768 | 5,768 | 0 | 0 |
| *Missing data were assessed using descriptive statistics. Most sociodemographic variables had complete observations. However, missing data were observed for birth interval (22.8%) and iron supplementation (9.5%). No imputation was performed, and complete-case analysis was applied in multivariable modelling.* | | | | |
