# Supplementary material for: Regional and socioeconomic inequalities and factors associated with low birth weight in Ghana: a multilevel analysis of the 2022 Ghana Demographic and Health Survey
Source: Front Pediatr. 2026 Oct 2;14:1846424. doi: 10.3389/fped.2026.1846424 (PMC13672600; doi:10.3389/fped.2026.1846424)
Supplement: Supplementary file 4 [file Table3.docx]

| **Supplementary File 3. Multivariate Mixed-Effects Logistic Regression Model of Determinants of Low Birth Weight (LBW) with Wealth–Residence Interaction** | | |
| --- | --- | --- |
| **Variables** | **AOR (95% CI)** | **P-value** |
| **Residence** |  |  |
| Urban | Ref |  |
| Rural | 2.30 (0.83–6.37) | 0.109 |
| **Wealth Index** |  |  |
| Richest | Ref |  |
| Poorer | 1.45 (0.50–4.23) | 0.501 |
| Middle | 2.38 (0.85–6.69) | 0.100 |
| Richer | 2.11 (0.74–6.03) | 0.164 |
| Richest | 1.14 (0.36–3.57) | 0.821 |
| **Residence × Wealth Interaction** |  |  |
| Rural × Poorer | 0.62 (0.20–1.96) | 0.418 |
| Rural × Middle | 0.25 (0.08–0.83) | **0.024*** |
| Rural × Richer | 0.24 (0.06–0.87) | **0.030*** |
| Rural × Richest | 0.71 (0.17–2.93) | 0.641 |
| **Maternal Age** |  |  |
| 15–19 years | Ref |  |
| 20–24 years | 1.36 (0.37–4.96) | 0.645 |
| 25–29 years | 1.81 (0.50–6.54) | 0.368 |
| 30–34 years | 1.66 (0.45–6.17) | 0.447 |
| 35–49 years | 1.77 (0.47–6.71) | 0.401 |
| **Ever Born Children** |  |  |
| 1 child | Ref |  |
| 2–3 children | 0.78 (0.44–1.38) | 0.392 |
| 4–5 children | 0.57 (0.30–1.11) | 0.098 |
| 6–7 children | 0.65 (0.31–1.37) | 0.258 |
| 8+ children | 0.40 (0.14–1.10) | 0.075 |
| **Birth Interval** |  |  |
| Short | Ref |  |
| Optimal | 0.71 (0.50–0.99) | **0.044*** |
| Long | 0.83 (0.55–1.26) | 0.391 |
| **Multiple Pregnancy** |  |  |
| Single | Ref |  |
| Multiple | 14.34 (9.59–21.41) | **<0.001*** |
| **ANC Visits** |  |  |
| Inadequate (<8 visits) | Ref |  |
| Adequate (≥8 visits) | 0.59 (0.44–0.80) | **0.001*** |
| **Maternal Education** |  |  |
| No education | Ref |  |
| Primary | 1.08 (0.69–1.67) | 0.742 |
| Secondary | 1.17 (0.80–1.71) | 0.415 |
| Higher | 1.55 (0.83–2.91) | 0.170 |
| **Working Status** |  |  |
| No | Ref |  |
| Yes | 0.95 (0.69–1.31) | 0.740 |
| **Marital Status** |  |  |
| Never married | Ref |  |
| Married | 0.66 (0.36–1.20) | 0.173 |
| Living with partner | 0.99 (0.54–1.81) | 0.962 |
| Widowed | 0.90 (0.18–4.49) | 0.898 |
| Divorced | 0.37 (0.04–3.13) | 0.360 |
| Separated | 0.50 (0.17–1.52) | 0.222 |
| **Religion** |  |  |
| Christian | Ref |  |
| Islam | 1.15 (0.81–1.62) | 0.436 |
| Traditional/Spiritualist | 0.87 (0.28–2.69) | 0.803 |
| No religion | 0.66 (0.24–1.81) | 0.422 |
| **Region** |  |  |
| Western | Ref |  |
| Central | 1.62 (0.67–3.89) | 0.284 |
| Greater Accra | 0.66 (0.36–1.81) | 0.419 |
| Volta | 0.67 (0.34–1.96) | 0.460 |
| Eastern | 1.46 (0.59–3.59) | 0.415 |
| Ashanti | 1.82 (0.91–4.19) | 0.162 |
| Western North | 0.97 (0.38–2.48) | 0.957 |
| Ahafo | 0.71 (0.36–1.88) | 0.493 |
| Bono | 1.37 (0.43–3.35) | 0.489 |
| Bono East | 1.42 (0.60–3.35) | 0.424 |
| Oti | 1.12 (0.40–2.80) | 0.813 |
| Northern | 2.54 (1.07–6.05) | **0.035*** |
| Savannah | 1.03 (0.41–2.60) | 0.950 |
| North East | 1.07 (0.43–2.68) | 0.883 |
| Upper East | 0.98 (0.39–2.49) | 0.972 |
| Upper West | 0.91 (0.35–2.36) | 0.849 |

*AOR = Adjusted Odds Ratio; CI = Confidence Interval; Ref = Reference category. Estimates were obtained from a multilevel mixed-effects logistic regression model accounting for clustering at the community level. The model included an interaction term between household wealth index and place of residence (wealth × residence). All variables shown in the table were adjusted for simultaneously. Statistical significance was set at p < 0.05;* *p < 0.05.*
